# Improved repair of rabbit calvarial defects with hydroxyapatite/chitosan/polycaprolactone composite scaffold-engrafted EPCs and BMSCs

**DOI:** 10.3389/fbioe.2022.928041

**Published:** 2022-08-03

**Authors:** Hedong Yu, Lingyun Xia, Xieyuan Leng, Yongji Chen, Li Zhang, Xiaobing Ni, Jie Luo, Weidong Leng

**Affiliations:** ^1^ Department of Stomatology, Taihe Hospital, Hubei University of Medicine, Shiyan, China; ^2^ Institute of Dental Research, School of Dentistry, Hubei University of Medicine, Shiyan, China; ^3^ The First Clinical College, Anhui Medical University, Hefei, China; ^4^ Department of Neurosurgery, Taihe Hospital, Hubei University of Medicine, Shiyan, China

**Keywords:** HA/CS/PCL composite scaffold, endothelial progenitor cells, bone marrow mesenchymal stem cells, calvarial defect, BMP-2, VEGF, PDGF

## Abstract

Endothelial progenitor cells (EPCs) expressing vascular endothelial growth factor (VEGF) and platelet-derived growth factor (PDGF) and bone marrow mesenchymal stem cells (BMSCs) expressing endogenous bone morphogenetic protein-2 (BMP-2) play the important role in new bone formation. This study investigated the effects of a porous hydroxyapatite (HA)/chitosan (CS)/polycaprolactone (PCL) composite scaffold-engrafted EPCs and BMSCs on the expression of BMP-2, VEGF, and PDGF in the calvarial defect rabbit model *in vivo*. It showed that a three-dimensional composite scaffold was successfully constructed by physical interaction with a pore size of 250 μm. The HA/CS/PCL scaffold degraded slowly within 10 weeks and showed non-cytotoxicity. By X-ray, micro-CT examination, and H&E staining, compared with the HA/CS/PCL group, HA/CS/PCL + EPCs, HA/CS/PCL + BMSCs, and HA/CS/PCL + EPCs + BMSCs groups performed a more obvious repair effect, and the dual factor group presented particularly significant improvement on the percentages of bone volume at week 4 and week 8, with evident bone growth. Osteogenesis marker (BMP-2) and vascularization marker (VEGF and PDGF) expression in the dual factor group were much better than those of the HA/CS/PCL control group and single factor groups. Collectively, the HA/CS/PCL composite scaffold-engrafting EPCs and BMSCs is effective to repair calvarial defects by regulating endogenous expression of BMP-2, VEGF, and PDGF. Thus, this study provides important implications for the potential clinical application of biomaterial composite scaffold-engrafted engineering cells.

## Introduction

High incidence diseases such as tumor, trauma, and infection contribute to different degrees of bone defect that cannot be healed by the natural recovery (Baroli, 2009). Biomaterials for bone tissue regeneration and repair are the frontier and hot issue of regenerative medicine research, and scaffold biomaterials are one of the key elements for bone tissue engineering. High biocompatibility, osteogenic ability, and biodegradation are of particular significance for the discovery of scaffold biomaterials. Therefore, there is still a great development for tissue-engineered bone in clinical application (Dumont et al., 2016; Kai et al., 2016).

Hydroxyapatite (HA) is similar to the inorganic components of calvaria in composition and structure, but it is fragile and easy to crack (Oliveira et al., 2017). Chitosan (CS) is a linear natural polysaccharide macromolecule, which has excellent biocompatibility, biodegradability, hydrophilicity, cell affinity, and adhesion (Tian et al., 2022). Polycaprolactone (PCL) is a linear polymer with high mechanical strength and easy processing, and its degradation products are easy to metabolize and eliminate (Lee et al., 2016). HA, CS, and PCL have been applied in bone repair for years. Natural bone tissue is mainly formed by the interaction of nanohydroxyapatite inorganic minerals and collagen organic matter in an orderly arrangement. In order to highly simulate the three-dimensional nanostructure of natural bone, our research group proposed the construction technology of three-component and porous bone composite scaffold containing PCL, CS, and HA and resolved the morphology control of the scaffold by 3D printing (Yu et al., 2020). The biomaterial showed good mechanical properties, bone conductivity, and induction, of which superior mechanical and biological properties displayed a good application prospect for bone defect repair (Gao et al., 2015). However, the biological characteristics of the HA/CS/PCL composite scaffold still need to be evaluated, biocompatibility, effectiveness in loading seed cells, and *in vitro* and *in vivo* osteogenic activity are needed to be tested.

As the seed cells for tissue engineering bone, bone marrow mesenchymal stem cells (BMSCs) are difficult to survive *in vivo*, so that the vascularization could be restricted, which is of decisive significance for bone regeneration and fusion (Stegen et al., 2015). Meanwhile, the endothelial progenitor cells (EPCs) extracted from the peripheral blood and BMSCs are co-cultured together to build a microenvironment in line with the physiological conditions of the human body, which could build the microvasculature by EPCs to supply the blood for the osteogenesis of tissue engineering bone, and promote the survival and osteogenesis of BMSCs (Goerke et al., 2015).

In this study, our research group intended to construct HA/CS/PCL scaffolds inoculating EPCs and BMSCs. In addition, the biocompatibility and bone repair effect of this composite were evaluated, and the effects of this composite on osteogenesis and vascularization using bone morphogenetic protein-2 (BMP-2), vascular endothelial growth factor (VEGF), and platelet-derived growth factor (PDGF) expression were measured as well.

## Materials and methods

### Reagents

The chitosan and hydroxyapatite were purchased from Solarbio (Beijing, China), polycaprolactone and simulated body fluid (SBF) from Sigma-Aldrich (Merck KGaA, Darmstadt, Germany), reagents used for cell culture all from Thermo Fisher Scientific (Loughborough, United Kingdom), and CCK-8 reagent from Beyotime (Shanghai, China).

### Microscopic examination

The HA/CS/PCL scaffold was observed using the Hitachi S-4300 cold field emission scanning electron microscope (Hitachi, Tokyo, Japan), and its morphology was analyzed with gold coating.

### X-ray diffraction study

X-ray diffraction (XRD) of the HA/CS/PCL scaffold was detected using D8 ADVANCE (BRUKER, Germany). JCPDS files 09-0432 were used to confirm the various peaks developed in HA.

### Immersion test

The *in vitro* immersion test was conducted to observe and evaluate the degradation behavior of scaffolds in SBF (M_SBF_: M_scaffold_ = 100:1), where the scaffolds were dried, weighed, immersed, and incubated with shaking at 100 rpm at 37°C. Every week one scaffold was randomly taken out to weigh the quality to analyze the degradation performance, and the degradation rate was calculated according to the equation, degradation rate = (M_0_ − M)/M_0_ × 100%, where M_0_ and M are the initial mass and mass at specific time point, respectively.

### Cytocompatibility tests

Immersed in DMEM containing 10% FBS (V_medium_: M_scaffold_ = 10 ml:1 g), the scaffold was incubated at 37°C for 120 h in a CO_2_ incubator, and then the medium was sterilized with a 0.22-μm micron bacteria-retentive filter to obtain the extracts. Moreover, for the cell viability test, the L929 cells were seeded in 96-well plates, with 2,000 cells/well. After overnight incubation, the medium was replaced with the HA/CS/PCL scaffold extracts (10, 30, 50, and 100%) for 2, 4, and 7 days cell culture. The test of cell viability was performed by CCK-8 kit, according to the manufacturer’s instructions.

### Animals

Twenty four subjects of this study, New Zealand rabbits, were purchased from Nanchang Longping Rabbit Industry Co., Ltd. with license No. scxk (GAN) 2014-0005. The ethics committee of Hubei University of Medicine approved the animal protocols.

### Isolation, culture, and immunophenotyping of BMSCs

After the removal of the femur and tibia, the bone marrow was extruded from the central canal of the bone, and the whole marrow cells were cultured with α-MEM supplemented with 10% fetal bovine serum and antibiotics. By removing the non-adherent cells after 24 h with the medium replacement, BMSCs were obtained and serially subcultured in the ratio of 1:3. BMSCs were used within four passages.

As the adherent cells reach 80%–90% confluence (generally about 10 days), BMSCs were stained with FITC-conjugated CD29 and PE-conjugated CD45 antibodies for 30 min at 4°C and washed with PBS. With at least 10,000 cells per sample analyzed, the expression of cell surface antigens CD29 and CD45 was detected by using a flow cytometer.

### Isolation, culture, and immunophenotyping of EPCs

The isolation of EPCs from the bone marrow was made by the lymphocyte separation solution as described previously (Ding et al., 2019). The isolated cells, incubated in a CO_2_ incubator at 37°C, were collected and stained with APC-conjugated CD133 antibody, PE-conjugated CD309 antibody, or PE-conjugated CD34 antibody. After staining for 30 min at 4°C in dark and washing with PBS, the utilization of the flow cytometer helped to detect the CD133, CD309, or CD34 expression of EPCs.

### Calvarial defect model

First, rabbits were anesthetized and fixed in the prone position, and they were trephine drilled into the anterior fontanelle with 1-cm diameter. Calvarial defects under copious saline irrigation with care to maintain the dura mater intact. Then, the composite scaffold with presence and absence of engrafted cells was placed in each defect, and the incision was sutured layer-by-layer and wrapped by the sterile dressing. After surgery, all animals were postoperatively cared by monitoring infection and diarrhea with subcutaneous injections of 0.1 mg kg^−1^ buprenorphine for 3 days and drinking water including trimethoprim–sulfamethoxazole. After 8 weeks, the specimens were harvested for the subsequent examination.

The rabbits were randomly divided into four groups (*n* = 6), namely, as the HA/CS/PCL scaffold group, HA/CS/PCL scaffold + EPCs group, HA/CS/PCL scaffold + BMSCs group, and HA/CS/PCL scaffold + EPCs + BMSCs group. Each piece of scaffold was soaked in cell suspension of the third-generation of BMSCs or EPCs with 0.5 × 10^6^/ml for 4 h in the CO_2_ incubator and before implantation, the scaffolds engrafted cells were washed by PBS. At the post-surgery of the 2nd, 4th, and 8th week, two rabbits (*n* = 2) from each group were randomly chosen for analysis.

### X-ray examination

As time progressed to weeks 2, 4, and 6 post-surgery, X-ray examination was performed by an X-ray machine (OEC9800, Shanghai Xianwei Optoelectronic Technology Co., Ltd.) to observe the repair of calvarial defects.

### Micro-CT

At the post-surgery of the 2nd, 4th, and 8th week, approximating images of calvaria with new bone deposition were reconstructed by three-dimensional micro-computerized tomography (micro-CT), whose scanning parameters are 80kV, 500 μA, and resolution of 27 μM, and the reestablishment of three-dimensional (3D) images and volumetric analysis were performed by the Inveon Research Workplace 2.2. Bone volume (BV) and tissue volume (TV) were used to quantify the calvaria with new bone, and their percentages of bone volume (BV/TV) of each graft site were measured.

### Hematoxylin and eosin (H&E) staining

After micro-CT imaging, the calvaria were decalcified for 5 weeks. The specimens of the calvarial defect region were dehydrated and embedded in paraffin and sectioned coronally with 5-μm thickness. The sections were stained with hematoxylin and eosin to visualize tissue structure.

### Western blot

The extraction of total protein from the bone tissues in the defect areas of the calvaria was performed by using the triplePrep kit (GE Healthcare Life Sciences), and its concentration was determined with a BCA assay kit. 25 μg protein per sample was separated *via* 10% SDS-PAGE and transferred to the PVDF membrane as described (Luo et al., 2021). After blocking in 5% skim milk at room temperature for 2 h, PVDF membranes were treated with the following primary antibodies overnight at 4°C, rabbit polyclonal anti-BMP-2 (1:1000, bs-1012R, Bioss), rabbit polyclonal anti-VEGF (1:1000, bs-1313R, Bioss), and rabbit polyclonal anti-PDGF (1:1000, bs-0196R, Bioss), followed by incubation with HRP-labeled IgG at 37°C for 2 h. The western blots were stained with an ECL reagent (Santa Cruz Biotech, CA, United States) and visualized by the ChemiDoc Touch Imaging System—Bio-Rad Laboratories (Bio-Rad Lab, Hercules, CA, United States).

### Statistical analysis

All the data were presented as mean and standard deviation and analyzed by SPSS 19.0. Overall significant differences among groups were determined by one-way ANOVA. When overall analysis demonstrated the presence of significant differences among all of the different groups studied, the differences between specific groups were tested using a *post-hoc* analysis, Tukey’s test. *p* < 0.05 was set as the threshold to indicate significant difference between groups.

## Results

### SEM ultrastructure analysis

**FIGURE 1:**
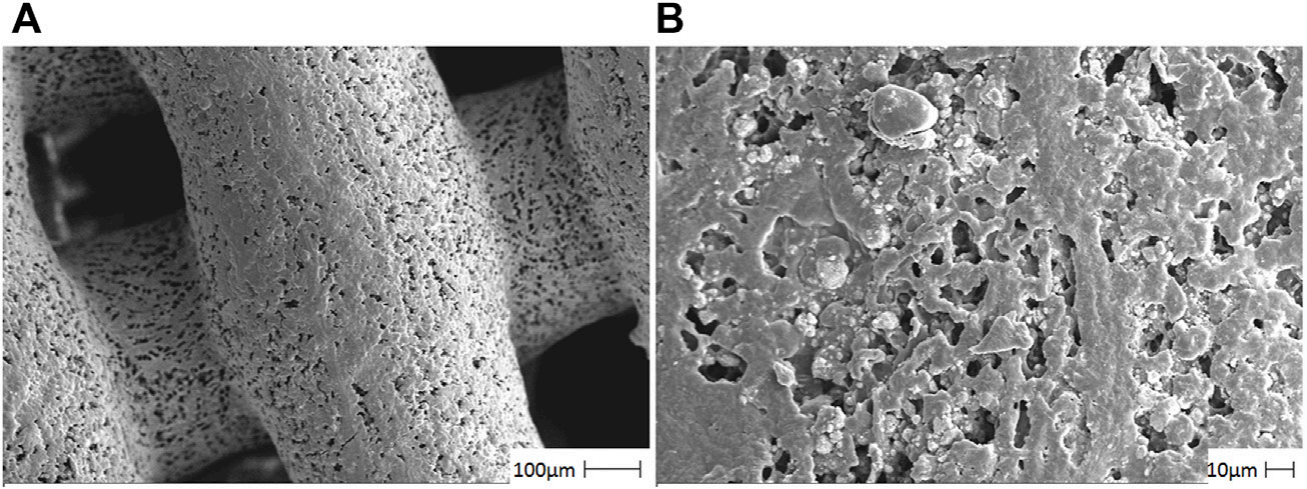
Photomicrographs of the HA/CS/PCL composite scaffold material by using a scanning electron microscope. The representative figures were observed on 100 µm **(A)** and 10 µm **(B)**, respectively.

In Figure 1, the HA/CS/PCL scaffold showed the neatly arranged fibers composed of CS and PCL in grid shape with an average pore size of about 250 μm. HA distributed evenly on the fiber surface in granular form, which proved that the ternary composite presented uniform and loose macroporous structure.

### XRD analysis

**FIGURE 2:**
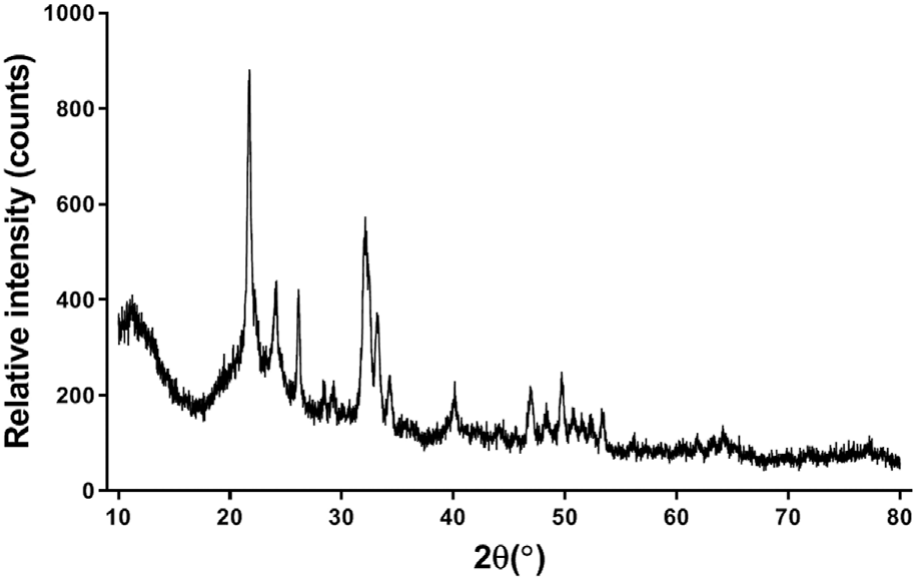
XRD spectra of the HA/CS/PCL composite scaffold.

For the HA/CS/PCL composite scaffold, peaks corresponding to HA crystalline phases appeared (Figure 2). The peaks of HA match well with JCPDS No. 09-0432. It indicated that the scaffold was combined with HA, CS, and PCL by physical interaction, which did not affect the biological function of HA.

### *In vitro* degradation behavior

**FIGURE 3:**
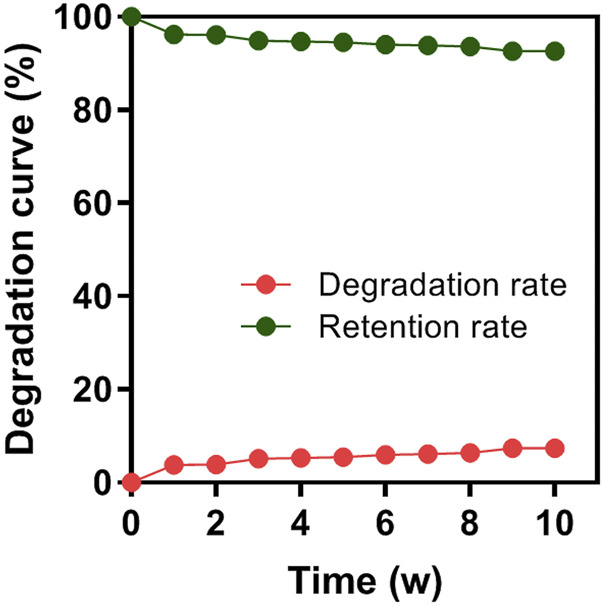
Degradation rate of the HA/CS/PCL composite scaffold in 1–10 weeks of immersion in simulated body fluid (SBF).

After 10 consecutive weeks, the degradation rate of the HA/CS/PCL composite scaffold in SBF was 7.39%, indicating that the HA/CS/PCL scaffold possessed certain mechanical stability and degraded slowly over time (Figure 3). This property meets the degradation and mechanical performance requirements of the biomaterial scaffold.

### *In vitro* cytocompatibility of HA/CS/PCL scaffold

**FIGURE 4:**
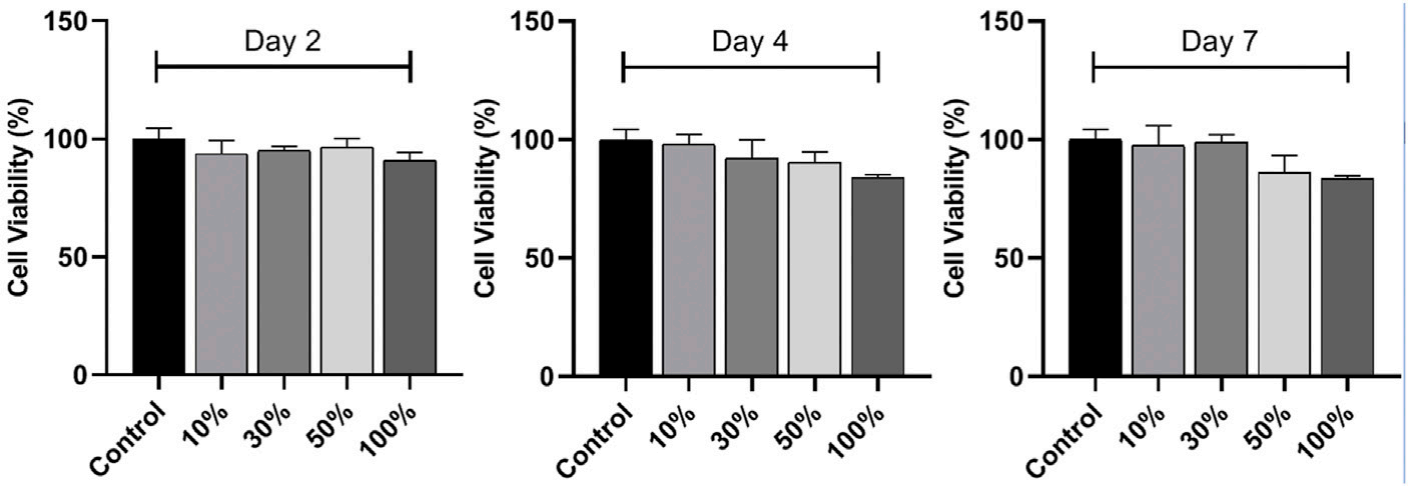
*In vitro* cytocompatibility of the HA/CS/PCL scaffold. CCK-8 assay of L929 cell proliferation on the HA/CS/PCL scaffold after 2, 4, and 7 days incubation. The data represent the mean ± SD.

L929 is the earliest and most widely used cell line in the cytotoxicity test because of its stable passage, rapid propagation, and low culture conditions *in vitro*. The cell viabilities were above 80% after 2, 4, and 7 days cell culture in the scaffold extracts compared with that of the blank control, indicating non-cytotoxicity and acceptable cytocompatibility of the scaffolds, according to the criteria for the evaluation of cell toxicity test recommended in GB/T16886.5-1997-ISO10993-5:1992 (Figure 4).

### Identification of BMSCs and EPCs

**FIGURE 5:**
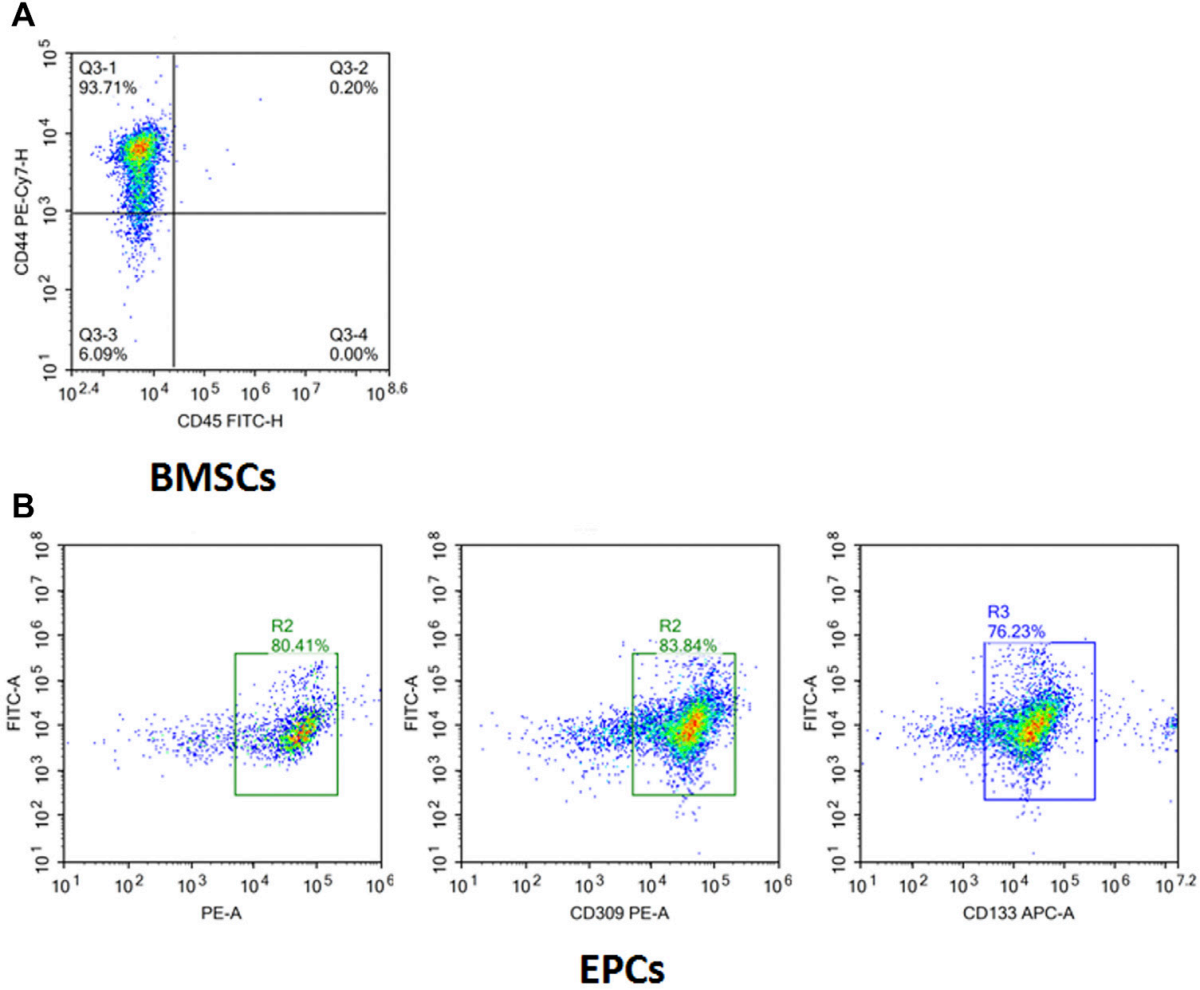
Surface markers of isolated and cultured BMSCs and EPCs. **(A)** Isolated cells were CD44^+^ (93.71%) and CD45^+^ (0.2%) determined by flow cytometry analysis. **(B)** Isolated cells were CD34^+^ (80.41%), CD309^+^ (83.84%), and CD133^+^ (76.23%) determined by flow cytometry analysis.

The flow cytometry results (Figure 5A) showed the cell surface antigen profile of BMSCs, which was identified that 93.71% of the cells expressed CD44, but was homogeneously negative for CD45 (0.2%). It confirmed the identification of isolated BMSCs. In addition to BMSCs, the isolated cells were positive for EPCs markers CD34 (80.41%), CD133 (83.84%), and CD309 (76.23%) (Figure 5B), which manifested that EPCs were successfully separated from rabbit bone marrow by the Ficoll density gradient method.

### Calvarial defect model and scaffold implantation

**FIGURE 6:**
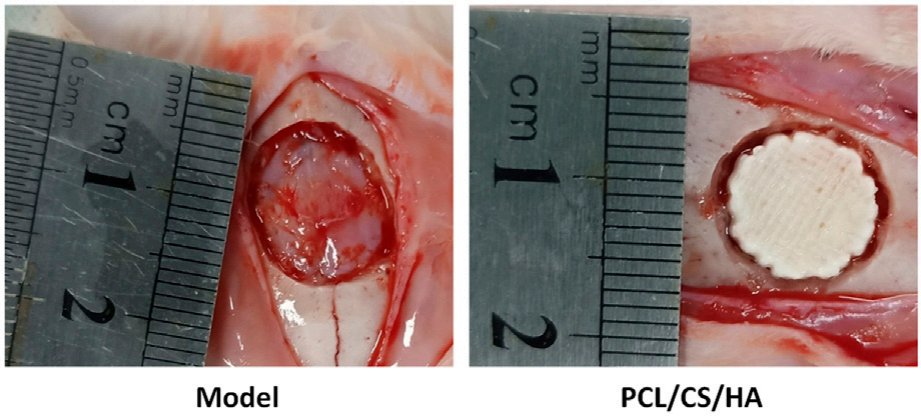
Representative images of the calvarial defect model (left) and HA/CS/PCL composite scaffold implantation (right).

As shown in Figure 6, after the composite scaffold was implanted into the calvaria, none of the rabbits died throughout the experiment, and all rabbits were under healthy conditions without infection or ulceration in the wound.

### EPCs and BMSCs improved the *in vivo* performance of HA/CS/PCL

**FIGURE 7:**
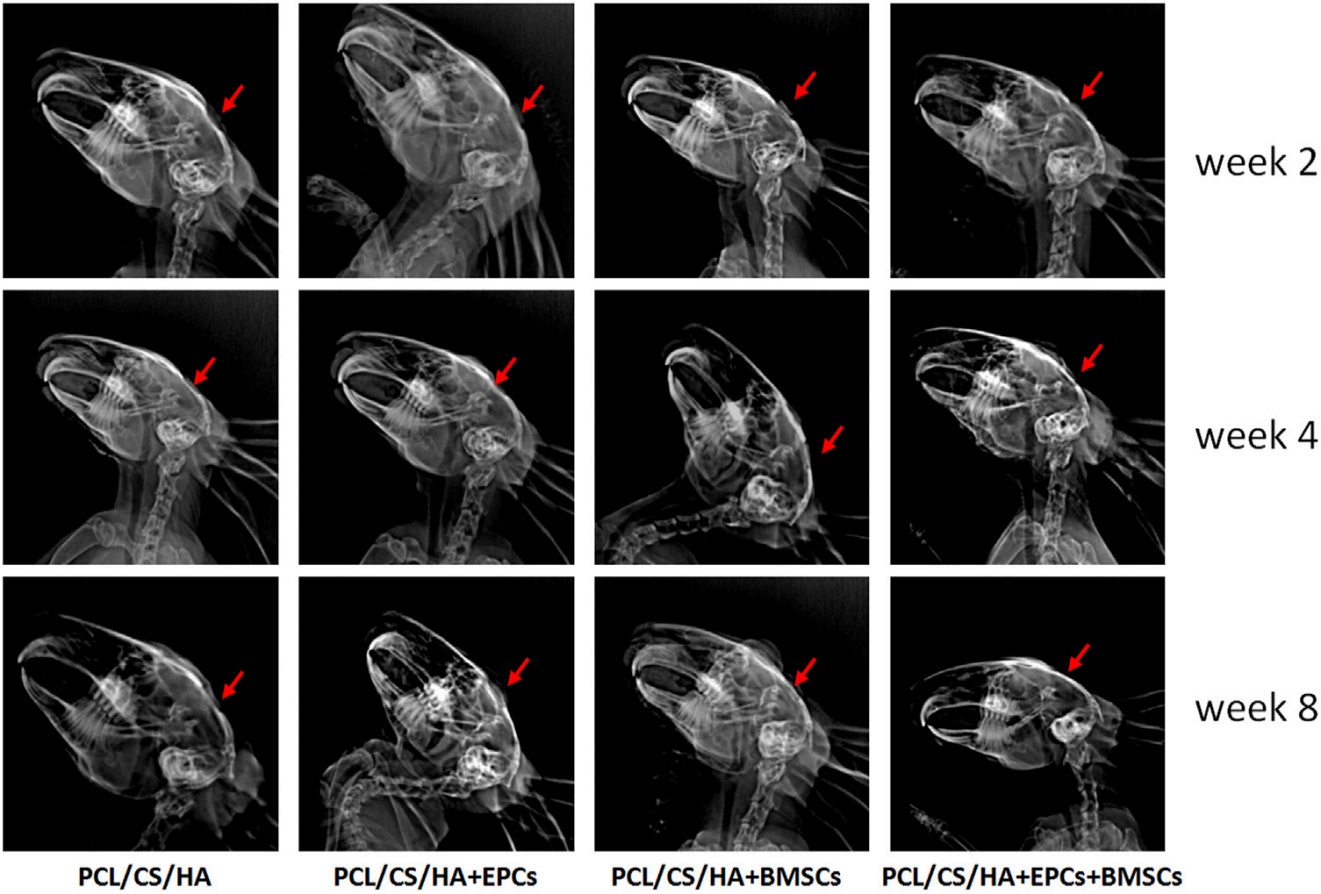
Evaluation of calvarial repair by X-ray images after 2, 4, and 8 weeks of implantation. The red arrow indicates the defect area receiving scaffold implantation.

As shown in Figure 7, HA/CS/PCL + EPCs, HA/CS/PCL + BMSCs, and HA/CS/PCL + EPCs + BMSCs, presenting obvious repair on the injured calvaria of rabbits, were more remarkable compared with HA/CS/PCL at the 8th week after scaffold implantation.

### EPCs and BMSCs accelerates HA/CS/PCL-based new bone formation

**FIGURE 8:**
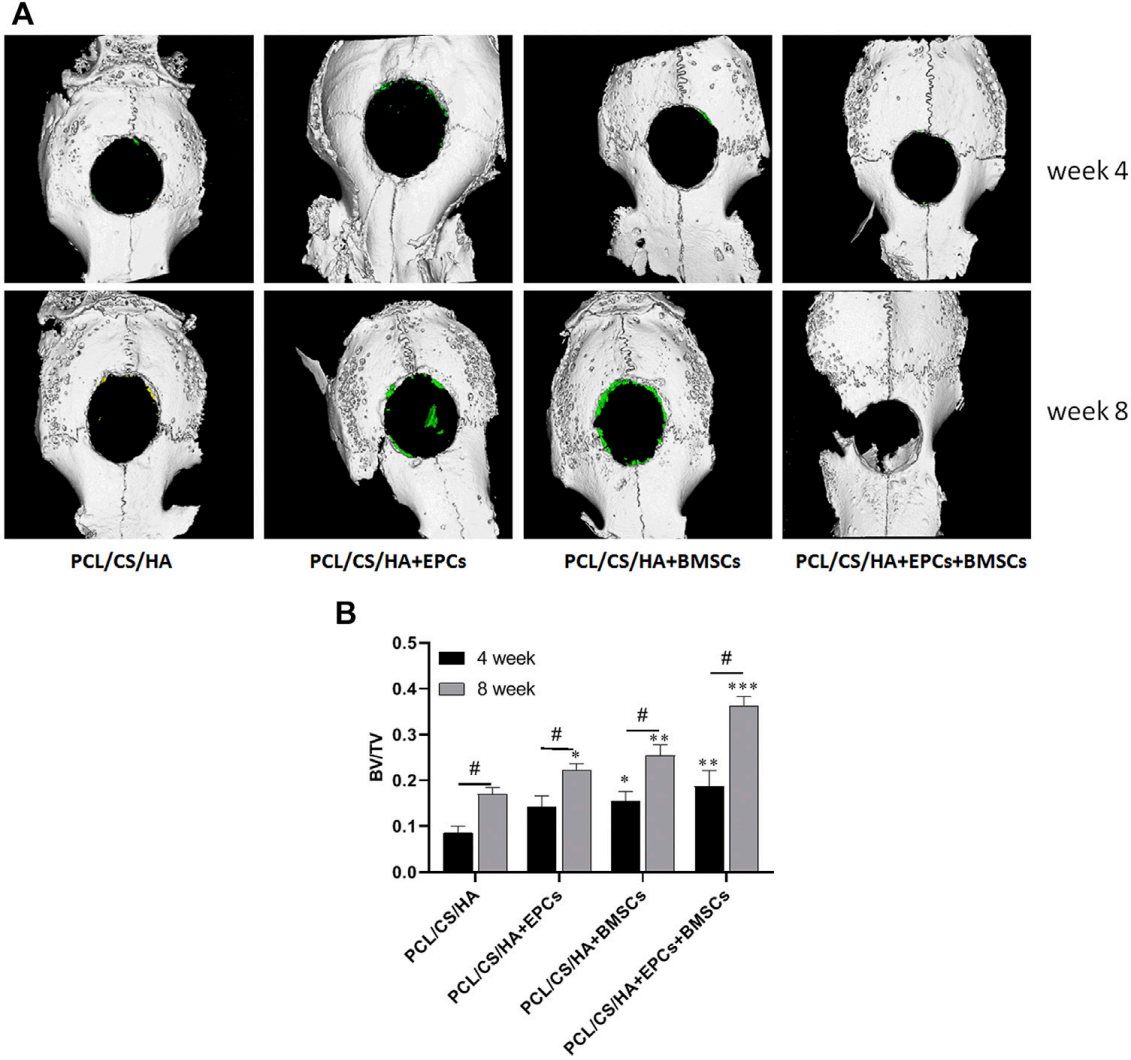
Three-dimensional micro-CT images of calvarial defect areas. The representative images **(A)** of micro-CT reconstruction and the quantitative data of BV/TV **(B)** from micro-CT evaluations of the four groups. *p < 0.05, **p < 0.01, ***p < 0.001 vs. HA/CS/PCL group (the same time point), #*p* < 0.05.

As shown in Figure 8A, compared with the HA/CS/PCL, BV/TV of HA/CS/PCL + EPCs, HA/CS/PCL + BMSCs, and HA/CS/PCL + EPCs + BMSCs groups increased significantly after 4 weeks and 8 weeks (*p* < 0.05), and the HA/CS/PCL + EPCs + BMSCs group presented the best calvarial repair effect at both the 4th and 8th week (Figure 8B).

### EPCs and BMSCs improves the histological repair results of calvarial defects

**FIGURE 9:**
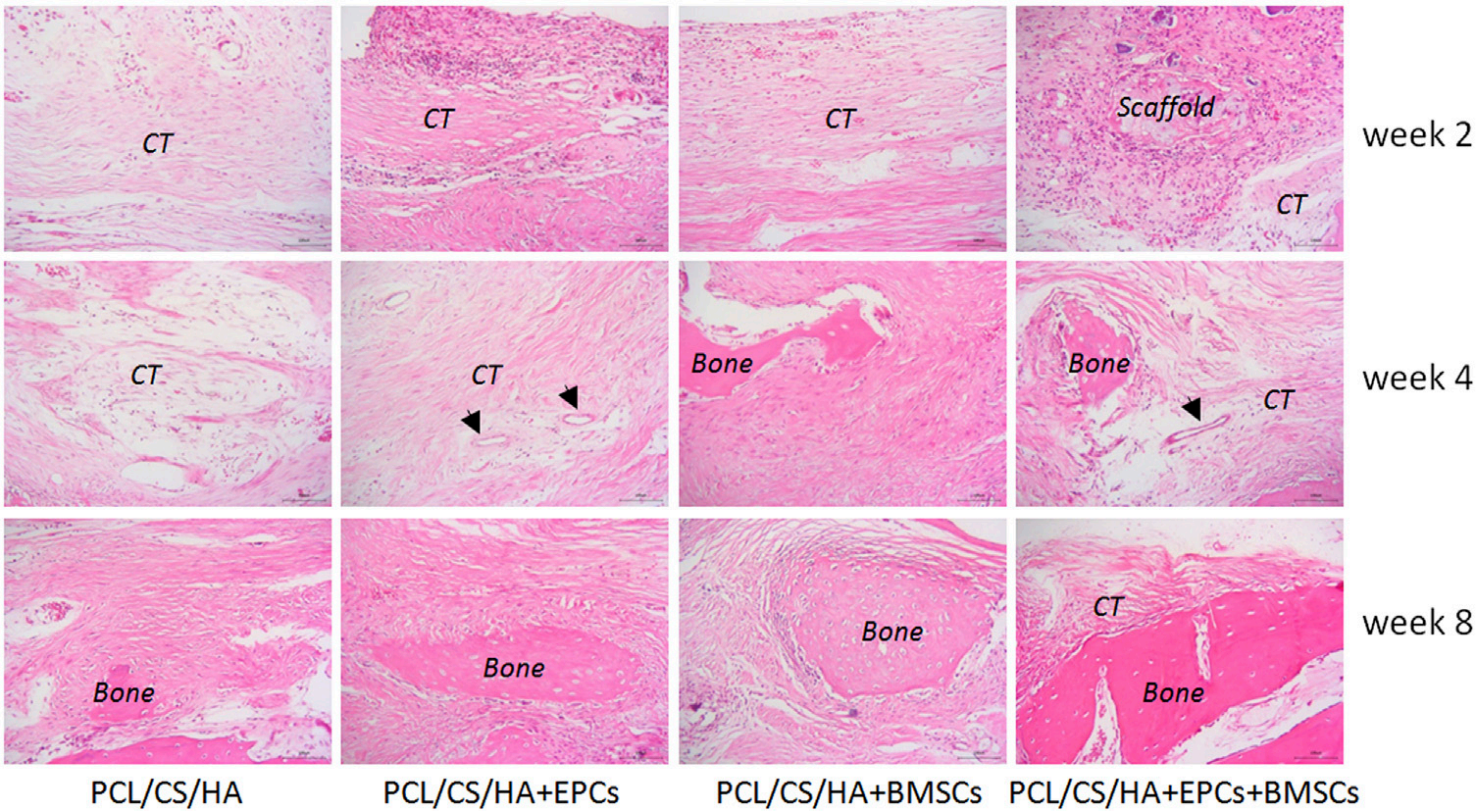
Histological structure of defected calvaria with composite scaffold repair at the post-surgery of the 2nd, 4th, and 8th week. New bone (bone), connective tissue (CT), and arrows indicate blood vessel.

As shown in Figure 9, by the 2nd week, all the groups failed to form bone after implantation. By the 4th week, connective tissue (CT) was found around the HA/CS/PCL composite scaffold, while there was significantly more CT in the HA/CS/PCL + EPCs + BMSCs group than those of the other three groups. New blood vessels could be found even in the HA/CS/PCL + EPCs and HA/CS/PCL + EPCs + BMSCs groups. By 8 weeks, new bone was observed in all the groups. Moreover, implantation of the composite scaffold of HA/CS/PCL, at calvarial defect site, performed moderate bone regeneration, but failed to completely repair the defects, while implantation of HA/CS/PCL + BMSCs and HA/CS/PCL + EPCs composite scaffolds, there were more new bone in the calvarial defect area, however, the HA/CS/PCL + EPCs + BMSCs composite scaffold resulted in the most significant repair of the defects.

### EPCs and BMSCs enhances the expression of BMP-2, VEGF, and PDGF

**FIGURE 10:**
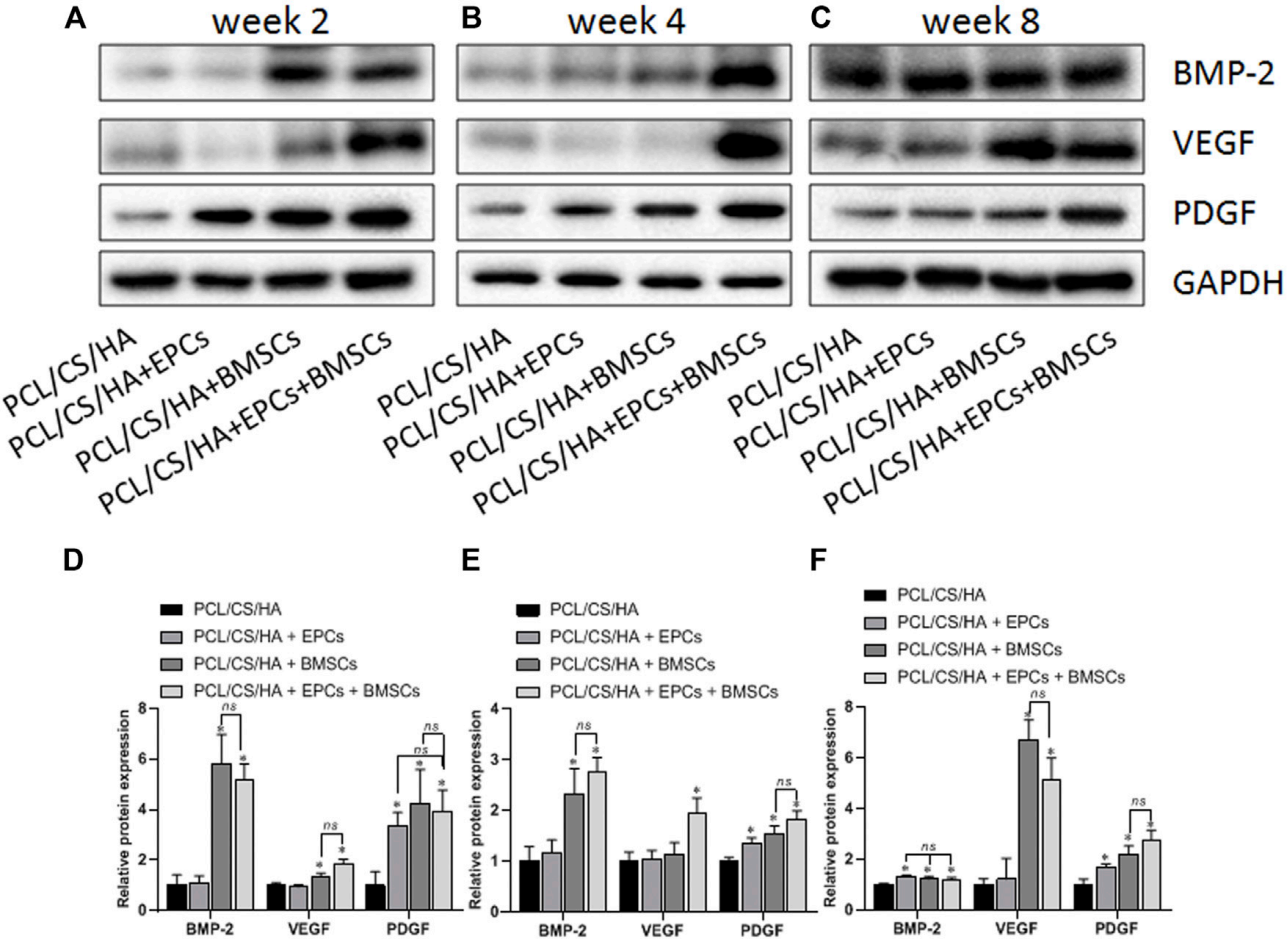
Expression levels of BMP-2, VEGF, and PDGF in the repaired calvaria with composite scaffold by western blot analysis. The representative bands and the quantified gray scale normalized to GAPDH for BMP-2, VEGF, and PDGF expression at the 2nd week **(A,D)**, 4th week **(B,E)**, and 8th week **(C,F)**. *p < 0.05 vs. HA/CS/PCL group, ns *p* > 0.05.

As shown in Figure 10, compared with the HA/CS/PCL group, BMP-2 expression in the HA/CS/PCL + BMSCs and HA/CS/PCL + EPCs + BMSCs groups was significantly higher at the 2nd and 4th week (*p* < 0.05). The HA/CS/PCL + EPCs, HA/CS/PCL + BMSCs, and HA/CS/PCL + EPCs + BMSCs groups all showed remarkably higher levels of BMP-2 at the 8th week compared with the HA/CS/PCL group (*p* < 0.05).

VEGF levels in the HA/CS/PCL + BMSCs and HA/CS/PCL + EPCs + BMSCs groups were greatly higher than that in the HA/CS/PCL group at the 2nd week (*p* < 0.05). At the 4th week, VEGF expression in the HA/CS/PCL + EPCs + BMSCs group exceeded that in the HA/CS/PCL group (*p* < 0.05). At the 8th week, VEGF expression in the HA/CS/PCL + BMSCs and HA/CS/PCL + EPCs + BMSCs groups were significantly higher than that in the HA/CS/PCL group (*p* < 0.05), which indicated potential neovascularization ability of HA/CS/PCL scaffolds engrafted BMSCs or EPCs.

The expression of PDGF in the HA/CS/PCL + EPCs, HA/CS/PCL + BMSCs, and HA/CS/PCL + EPCs + BMSCs groups increased remarkably at the 2nd, 4th, and 8th week compared with the HA/CS/PCL group (*p* < 0.05).

## Discussion

In this study, the composite scaffolds engrafted EPCs and BMSCs were developed, of which function in the calvarial repair was confirmed. Moreover, this study, providing the new experimental evidence for the treatment of calvarial repair, presented that the HA/CS/PCL scaffold possessed a mild repair effect, while the inoculation with EPCs and BMSCs obviously promoted the calvarial repair.

It has been found in our previous study that BMSCs possess the ability of directional migration by microphotography, which lays the foundation for the construction of tissue-engineered bone in this present study (Yang et al., 2014). Niemeyer et al. (2010) reported that human mesenchymal stem cells were transplanted into the critical-size bone defect of rabbit to examine the regeneration potential. Zong et al. (2010) evaluated the osteogenic effect of BMSCs and osteoblast-like cells combined with poly lactic-co-glycolic acid (PLGA) on the calvarial defect of rats and revealed that BMSCs were superior to osteoblasts in bone reconstruction. In this study, BMSCs and EPCs were isolated and identified on the basis of the surface markers and were used as seed cells, and then the reconstruction effects were evaluated on the calvarial defect of rabbits by BMSCs, EPCs, or combined BMSCs and EPCs in HA/CS/PCL scaffolds.

Scaffold material plays a crucial role in bone tissue engineering not only in supporting and maintaining the shape of original bone tissue but also as a biomaterial template providing attachment for engrafted cells to live, grow, and differentiate, and guiding the regeneration of damaged tissue and controlling the regenerated structure (Szpalski et al., 2012). In this study, the HA/CS/PCL porous ternary composite scaffold was prepared by three-dimensional printing and molding technology. As the scaffold was effectively solved by the three-dimensional and controllable internal and external structures, the obtained multi-scale structure and shape of scaffolds could be used to simulate the ideal bone repair material. Some studies have shown that scaffold using calcium-deficient hydroxyapatite and BMP-2–loaded sulfate chitosan could repair rat calvarial defect and promote new bone formation *in vivo* (Zhao et al., 2012). Chen et al. (2013) used macroporous calcium phosphate seeded with human umbilical cord mesenchymal stem cells to repair 8-mm rat cranial defects, which promoted new bone regeneration. In this study, X-ray, micro-CT, and pathological results manifested that HA/CS/PCL + EPCs, HA/CS/PCL + BMSCs, and HA/CS/PCL + EPCs + BMSCs groups had remarkable repair effect on bone defects. Particularly, at the 8th week, HA/CS/PCL + EPCs + BMSCs scaffold implantation performed more significantly. These results suggest that BMSCs may be combined with EPCs as seed cells, which possess neovascularization ability, to exert both the directional migration ability of BMSCs and the vascularization ability of EPCs to facilitate new bone formation.

The differentiation of BMSCs into osteoblast-like cells, osteogenesis induction by BMP-2 is of importance for improving the osteoinduction and osteoconduction properties of bone scaffold materials (Yamaguchi et al., 1996; Partridge et al., 2002; Li et al., 2006). The new bone formation by scaffold materials promotes vascularization, depending on the participation of growth factors, such as VEGF, PDGF (Nguyen et al., 2012), and transforming growth factor β (TGF-β) (Thurston, 2002). BMP is the main signaling pathway in osteogenic differentiation to boost the formation of new bone (Liu et al., 2017). As the strongest member in bone induction in the BMP family (Sun et al., 2017), BMP-2 can promote bone formation by direct application or local application in carriers (Demiray et al., 2017; Khorsand et al., 2017; Kuttappan et al., 2018) and enhance the expression of Runx2, a key transcription factor in bone differentiation, thus to activate the osteogenic process (Li et al., 2017). VEGF is recognized as the most powerful and specific angiogenic factor (Ju et al., 2018), which regulates bone formation by stimulating the differentiation and proliferation of osteoblasts, accelerating the formation and remodeling of bone (Huang et al., 2016). Both BMP-2 and VEGF play the important roles in bone regeneration as osteogenic factors and angiogenic factors, respectively (Kempen et al., 2009). It is reported that the expression of BMP-2 and VEGF enhance each other in the fracture healing (Zhang et al., 2011). VEGF could upregulate the expression of BMP-2 in osteoblasts and accelerate bone healing (Samee et al., 2008). In the initial steps of bone regeneration, PDGF promotes mitosis and angiogenesis, increases the synthesis of collagen and strengthens the local stress (Yang et al., 2017). Nash et al. (1994) injected collagen-containing rhPDGF into rabbits that underwent unilateral tibial osteotomy and found that rhPDGF increased the callus density, callus volume, and osteogenic differentiation. This present study showed that the expression of BMP-2, VEGF, and PDGF in the HA/CS/PCL + EPCs + BMSCs group increased at the 2nd, 4th, and 8th weeks, and there was a clear improvement in new bone formation and pathological change compared with the other three groups, which suggest that the better effect of HA/CS/PCL + EPCs + BMSCs on repairing bone defects may be related to the significantly increased expression of BMP-2, VEGF, and PDGF and their additive effect on bone regeneration.

In conclusion, this study proves that HA/CS/PCL + EPCs + BMSCs possess a better bone repair effect in the rabbit calvarial injury model, likely through increasing the expression of BMP-2, VEGF, and PDGF and, simultaneously, provides the experimental evidence for the future clinical application of the cell–matrix composite scaffold.

## Data Availability

The original contributions presented in the study are included in the article/Supplementary Material; further inquiries can be directed to the corresponding authors.
